# When is neoadjuvant chemotherapy indicated in rectal neuroendocrine tumors? An analysis of the National Cancer Database

**DOI:** 10.1007/s10151-024-02927-1

**Published:** 2024-05-21

**Authors:** R. Gefen, S. H. Emile, N. Horesh, Z. Garoufalia, M. R. Freund, S. D. Wexner

**Affiliations:** 1 Ellen Leifer Shulman and Steven Shulman Digestive Disease Center, Cleveland Clinic Florida, 2950 Cleveland Clinic Blvd., Weston, FL 33331 USA; 2 Department of General Surgery, Hadassah Medical Organization and Faculty of Medicine, Hebrew University of Jerusalem, Jerusalem, Israel; 3 Colorectal Surgery Unit, Mansoura University Hospital, Mansoura University, Mansoura, Egypt; 4 Department of Surgery and Transplantations, Sheba Medical Center, Ramat Gan, Israel; 5 Department of General Surgery, Shaare Zedek Medical Center, Faculty of Medicine, Hebrew University of Jerusalem, Jerusalem, Israel

**Keywords:** Neoadjuvant chemotherapy, Rectal neuroendocrine tumor, National Cancer Database, Surgical resection

## Abstract

**Background:**

Rectal neuroendocrine tumors (rNET) are rare and challenging to manage. While most patients with small rNET can be definitively treated with local excision, the role of chemotherapy in general and neoadjuvant therapy particularly in managing advanced rNET has not been well established. Therefore, this study aimed to determine which patients with rNET may gain a survival benefit from neoadjuvant chemotherapy.

**Methods:**

A retrospective cohort analysis of all patients who underwent surgical resection of rNET in the US National Cancer Database (NCDB) (2004–2019) was performed. First, univariate and multivariate Cox regression analyses were performed to determine the independent predictors of poor overall survival (OS) and define the high-risk groups. Afterward, stratified OS analyses were performed for each high-risk group to assess whether neoadjuvant chemotherapy had a survival benefit in each group.

**Results:**

A total of 1837 patients (49.8% female; mean age 56.6 ± 12.3 years) underwent radical resection of a rNET. Tumors > 20 mm in size, clinical T4 tumors, poorly differentiated tumors, and metastatic disease were independent predictors of worse OS and were defined as high-risk groups. Neoadjuvant chemotherapy did not have a significant survival benefit in any of the high-risk groups, except for patients with high-grade rNETs where neoadjuvant therapy significantly improved OS to a mean of 30.9 months compared with 15.9 months when neoadjuvant therapy was not given (*p* = 0.006).

**Conclusions:**

Neoadjuvant chemotherapy improved the OS of patients with high-grade rNET by 15 months and may be indicated for this group.

**Supplementary Information:**

The online version contains supplementary material available at 10.1007/s10151-024-02927-1.

## Introduction

The gastrointestinal (GI) tract is the most common site for neuroendocrine tumors (NETs), which are usually indolent in nature. Within the GI tract, the rectum is one of the most common sites for NETs, with an increasing incidence rate [1, 2]. However, this increased incidence is possibly due to the increasing use of endoscopy for screening and diagnosis, which has led to increased detection of early localized lesions [2, 3]. Despite the increase in the incidence of rectal NETs (rNETs), they remain rare, accounting for only 1–2% of all rectal tumors. [2]

Most rNETs are asymptomatic and usually found during screening colonoscopy. However, rNETs may present like other symptomatic rectal tumors with bleeding, bowel habit changes, and pain. Carcinoid syndrome is rare with rNETs [1, 4]. Staging mainly depends on the depth of invasion of rNETs and tumor size. Lymph node (LN) staging is based on the presence of tumor metastasis even in a single node [5].

Surgical resection is generally the initial therapy for rNETs. The extent of resection is generally based on tumor size, [5, 6], which is an important prognostic factor. Most rNETs are typically small and of low-to-intermediate grade [1]. Local resection is deemed safe in lesions up to 1 cm, [7] whereas radical resection with mesorectal clearance is the appropriate approach for tumors > 2 cm or with adverse features. There is an ongoing debate on the oncologic safety of local excision for tumors measuring between 1 and 2 cm.

Prior studies have demonstrated that tumor size and depth of invasion are predictors of LN metastasis [8]. Tumors > 1 cm, invasion of muscular layers, and lymphovascular invasion are all independent risk factors for metastatic disease [7]. Disease stage is also a significant survival predictor [9]. Survival of patients with NET has improved over time and is significantly correlated with age, race, disease stage, and site [3]. Overall survival (OS) depends on disease extent, grade, and site, [3] with a median recorded survival duration of 24.6 years [3].

As advanced disease is rare, the role of chemotherapy and radiotherapy for rNETs in general, and as neoadjuvant treatments specifically, has not been well established. There is a paucity of data on the impact of chemotherapy on the outcomes of patients with rNETs who undergo resection. According to the European Neuroendocrine Tumors Society (ENETS), chemotherapy should be considered in T4 and G2 or G3 tumors and when LN involvement is evident [1]. This study aimed to determine which patients with rNETs may gain a survival benefit from neoadjuvant chemotherapy.

## Patients and methods

Given the rarity of rNETs, we needed to assess a large number of patients, thus we used the National Cancer Database (NCDB) for the period 2004 to 2019. The NCDB includes hospital registry data from more than 1500 hospitals in the USA. The NCDB is a joint project of the Commission on Cancer (CoC) of the American College of Surgeons and the American Cancer Society. The de-identified data used in the study are derived from the NCDB and its participating hospitals are not responsible for the statistical validity of the analysis or the conclusions of the study. The study was a retrospective review of a public database that entails de-identified patients’ data, thus formal ethics approval and written consent to participate in the study were not required according to our institutional review board.

### Study population

Patient data were interpreted according to the NCDB Participant User File (PUF) dictionary. Patients with rNETs [International Classification of Diseases for Oncology-3 (ICDO-3) codes 8240/3, 8246/3, 8013, 8040, 8041,8249, 8241, 8243, 8245, 8574] in the NCDB who underwent radical resection (including low anterior resection, abdominoperineal resection, nonspecified proctectomy, and pelvic exenteration) were included in the study. We excluded patients who did not undergo surgery or who underwent local excision.

### Data collection

We collected demographic data (age, sex, race, Charlson–Deyo score, residence area, and insurance status), clinical and pathological tumor characteristics [TNM stage, number of harvested and involved lymph nodes (LN), tumor grade, lymphovascular invasion], and treatment approach (type of surgery, receipt of chemotherapy, radiotherapy or immunotherapy, or sequencing of therapy). The primary outcome of the study was OS.

### Study strategy

The study entailed a two-step approach. In the first step, we used univariate and multivariate Cox regression analyses to determine the independent predictors of poor survival after proctectomy of rNETS. Patients who had any of these predictive risk factors were defined as high-risk groups. Factors included in the regression analyses were selected based on previous literature and included age, sex, race, tumor size, clinical T stage, nodal status, clinical M stage, and tumor grade. In the second step, we performed stratified survival analyses for each high-risk group using the Kaplan–Meier test to determine which group may have a survival benefit after neoadjuvant chemotherapy.

### Statistical analysis

Statistical analyses were performed using EZR (version 1.55) and R software (version 4.1.2) [10] and SPSS (version 23). Continuous data were expressed as interquartile range (IQR). A student *t*-test or Mann–Whitney test was used to analyze continuous variables. Categorical data were expressed as numbers and percentages and were analyzed with the Fisher’s exact test or Chi-square test. We used Cox proportional hazard regression to evaluate risk factors. Kaplan–Meier statistics and log-rank test were used to detect differences in OS between the groups.

## Results

### Characteristics of the cohort

**Fig. 1:**
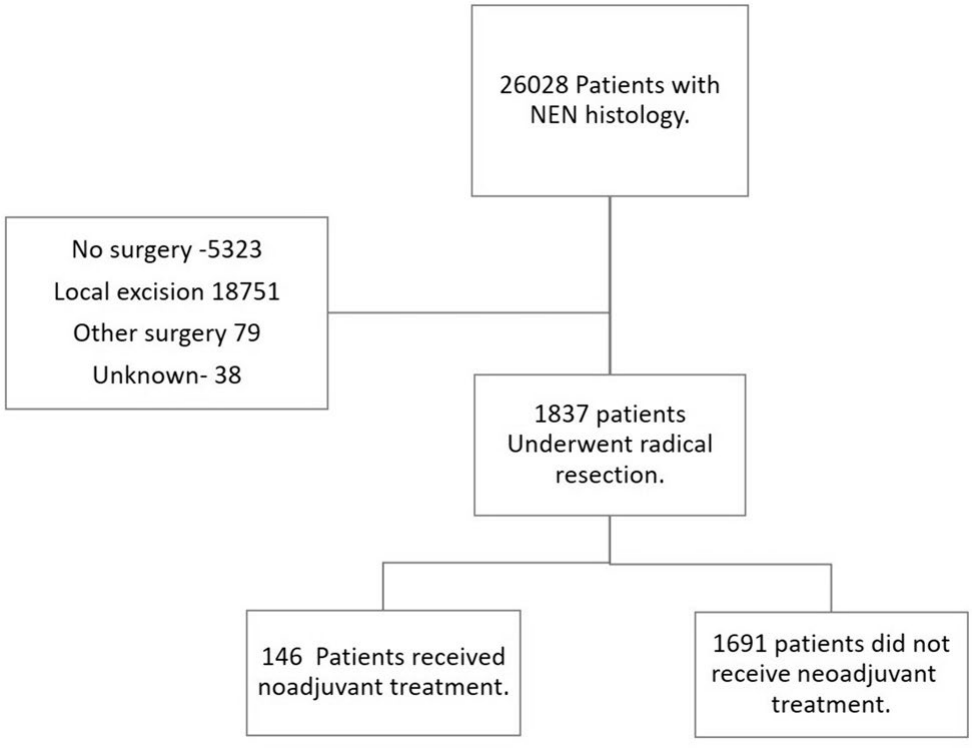
Flow chart for patient inclusion in the study

After screening the records of 26,028 patients with rNETs between 2004 and 2019, 1873 patients met the inclusion criteria and were included in the analysis (Fig. 1). Female patients accounted for 49.8% of the cohort and the mean age of patients was 56.6 ± 12.7 years. Two hundred fifty-six (13.7%) tumors were poorly or undifferentiated, while 39.3% were well or moderately differentiated; the grade was not available for 47% of patients. Overall, 414 (22.1%) tumors were > 20 mm in size, 191 (10.2%) had nodal involvement, and 120 (6.4%) had metastatic disease on clinical assessment. Chemotherapy was administered to 278 (14.8%) patients, 116 (41.7%) of whom received neoadjuvant chemotherapy, 132 (47.5%) received adjuvant treatment, and 30 (10.8%) received both. Details of chemotherapy regimens were not available. Of the patients who received chemotherapy, 163 (58.6%) also received radiation therapy either before or after surgery (see patient characteristics presented in Supplementary Table 1).

Patients with high-grade tumors were more often older, male, had a higher Charlson–Deyo score, presented more frequently with larger tumors and tumors of advanced T and N stage compared with patients with well or moderately differentiated tumors. Additionally, patients with high-grade tumors underwent abdominoperineal resection more often and were more likely to have positive surgical margins than patients with well or moderately differentiated tumor (Supplementary Table 2).

### Outcomes

On univariate analysis, sex, age, Charlson–Deyo score, tumor grade, clinical T stage, positive nodal status, and metastatic disease were significantly associated with OS. Tumor size was also significantly associated with OS as tumors < 10 mm had a hazard ratio (HR) of 0.36 (*p* < 0.001) and tumors > 20 mm had a HR of 3.46 (*p* < 0.001) (Table 1). On multivariate analysis, the independent predictors of poor OS were high-grade tumors, metastatic disease, and T4 tumors (Table 2). Low-grade tumors and those < 10 mm were predictors of better OS (Table 2).

**Table 1:** Univariate cox regression analysis

Variant	OS (%)	HR	Lower CI 95%	Upper CI 95%	*p*-Value
Patient related factors					
Age		1.057	1.05	1.06	** < 0.001**
Sex					
Female	75.8	Reference			
Male	67.3	1.5	1.25	1.78	** < 0.001**
Race					
American Indian	77.8	Reference			
Asian	83.6	0.78	0.18	3.3	0.731
Black	73.0	1.2	0.3	4.92	0.784
White	68.7	1.5	0.37	6.0	0.5702
Charlson–Deyo score					
0	74.8	0.63	0.5	0.8	** < 0.001**
1	64.5	Reference			
2	40.3	1.86	1.27	2.7	**0.0014**
3	46.4	1.86	1.08	3.2	**0.025**
Tumor characteristic factors					
Tumor grade					
Well/moderately differentiated	81.0	Reference			
Poorly/undifferentiated	28.1	6.85	5.48	8.57	** < 0.001**
Clinical T stage					
T1	84.1	Reference			
T2	66.0	2.44	1.6	3.7	** < 0.001**
T3	44.4	5.02	3.6	6.93	** < 0.001**
T4	25.7	10.38	6.57	16.4	** < 0.001**
Metastatic disease	31.1	3.7	2.9	4.76	** < 0.001**
Positive nodal status	46.6	3.739	2.9	4.8	** < 0.001**
Tumor size					
< 10 mm	89.4	0.36	0.25	0.53	** < 0.001**
10–20 mm	73.5	Reference			
> 20 mm	35.9	3.46	2.6	4.57	** < 0.001**

Bold text in the *p*-value column indicates statistical significance

*OS* Overall survival, *HR* hazard ratio, *CI* confidence interval

**Table 2:** Multivariate Cox regression analysis

Variant	HR	Lower CI 95%	Upper CI 95%	*p*-Value
Age	1.01	0.99	1.03	0.091
Sex				
Female	Reference			
Male	1.1	0.76	1.61	0.59
Charlson–Deyo score				
0	0.86	0.53	1.4	0.55
1	Reference			
2	1.63	0.74	3.6	0.22
3	0.53	0.06	4.1	0.54
Tumor grade				
Well/moderately differentiated	Reference			
Poorly/undifferentiated	2.77	1.77	4.33	** < 0.001**
Tumor size				
< 10 mm	Reference			
10–20 mm	3.45	1.44	8.3	**0.005**
> 20 mm	11.33	5.2	24.6	** < 0.001**
Clinical T stage				
T1	Reference			
T2	0.68	0.38	1.2	0.19
T3	0.67	0.37	1.19	0.173
T4	2.67	1.23	5.79	**0.012**
Positive nodal status	1.2	0.77	1.88	0.4
Metastatic disease	2.0	1.22	3.52	**0.006**

Bold text in the *p*-value column indicates statistical significance

*HR* Hazard ratio, *CI* confidence interval

**Fig. 2:**
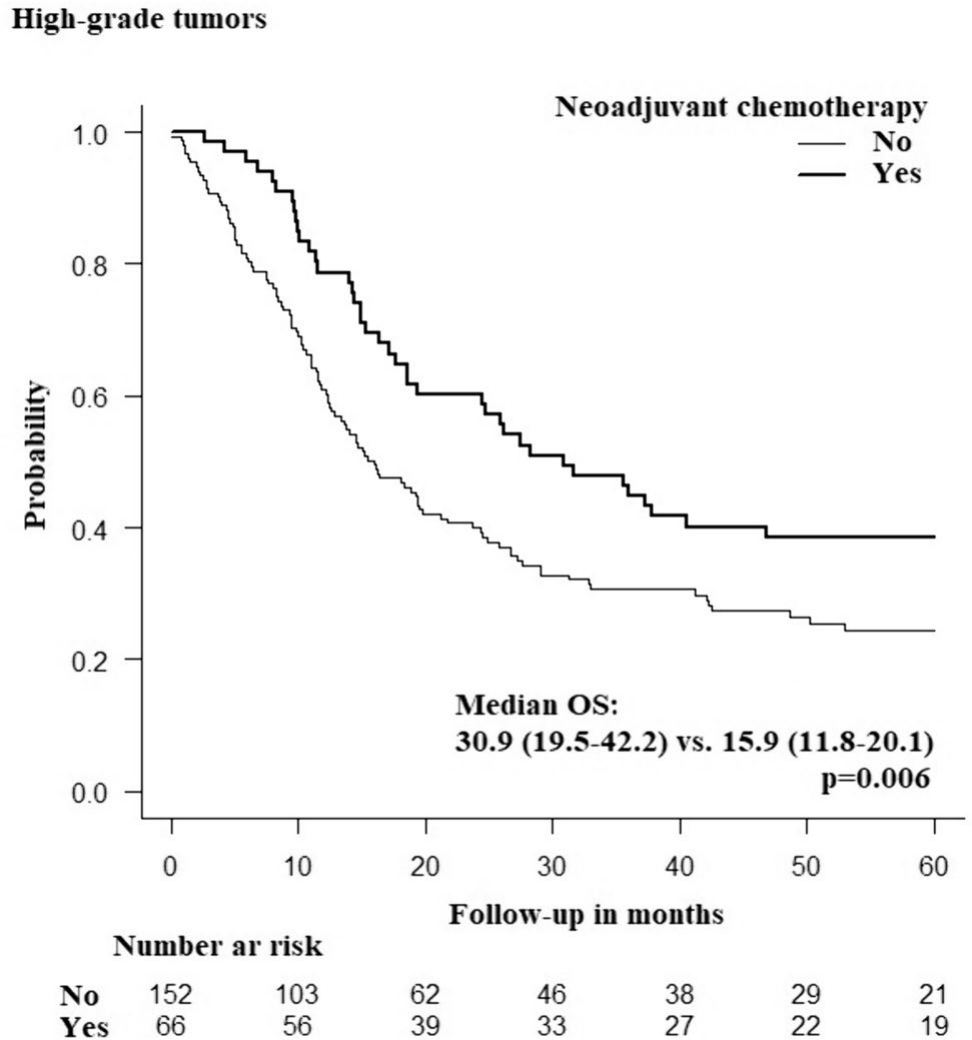
Kaplan–Meier curve showing difference in overall survival in high grade rectal neuroendocrine tumors according to the receipt of neoadjuvant chemotherapy

**Fig. 3:**
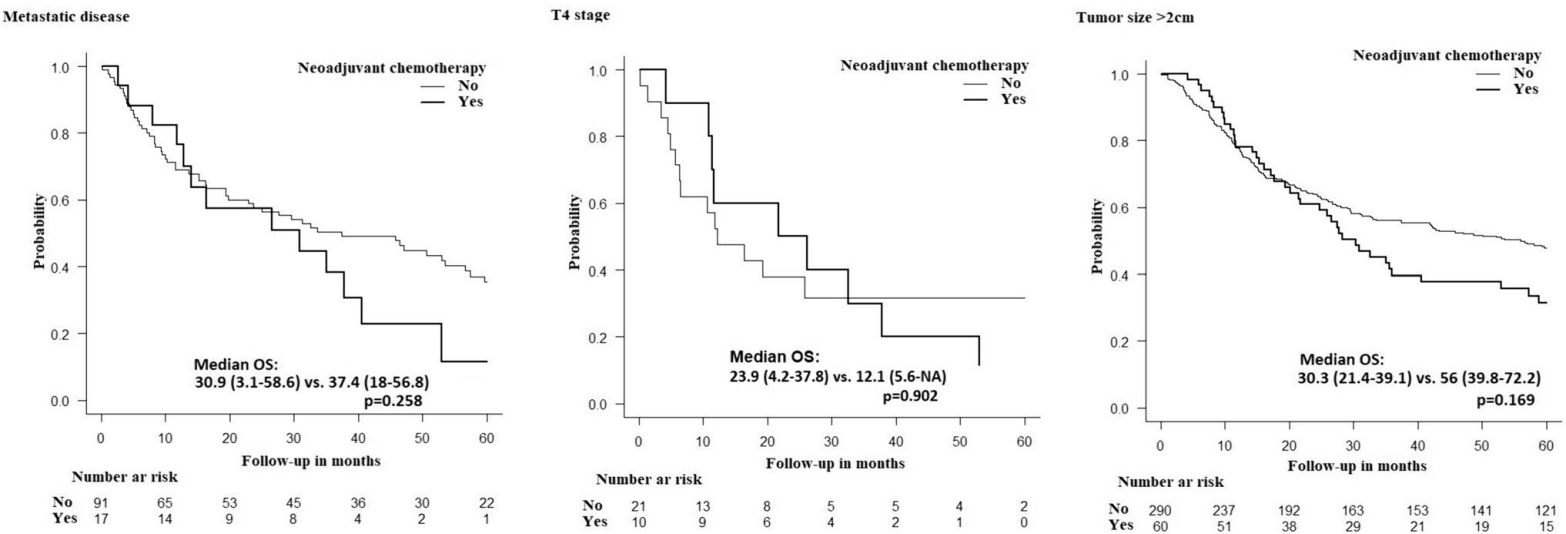
Kaplan–Meier curve showing difference in overall survival in patients with metastatic, T4, and > 2 cm rectal neuroendocrine tumors according to the receipt of neoadjuvant chemotherapy

**Fig. 4:**
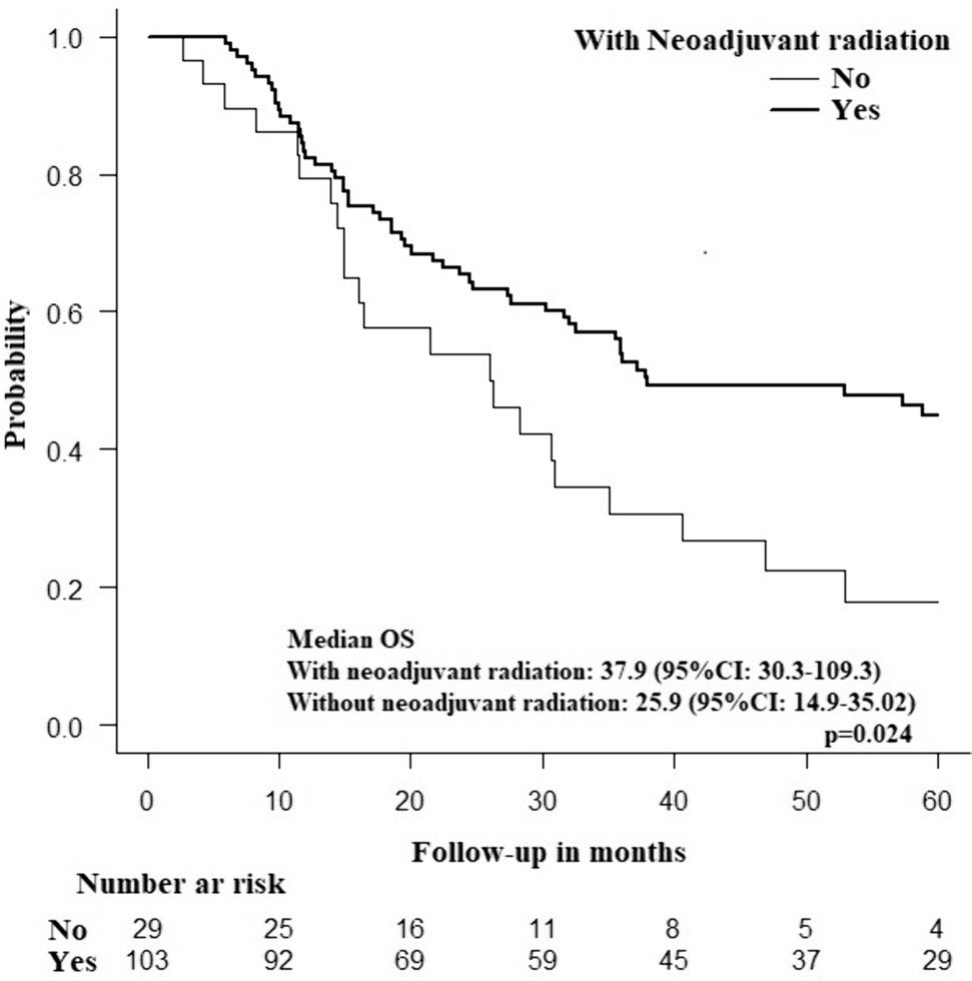
Kaplan–Meier curve showing difference in overall survival in rectal neuroendocrine tumors according to the receipt of neoadjuvant chemotherapy with and without neoadjuvant radiation therapy

Using the Kaplan–Meier test, patients with high-grade tumors treated with neoadjuvant chemotherapy had significantly longer median OS than did patients who did not receive neoadjuvant chemotherapy (30.8 versus 15.9 months; *p* = 0.006) (Fig. 2). No significant survival benefits of neoadjuvant treatment were noted for other high-risk groups, including tumors > 20 mm, T4 tumors, and metastatic disease (Fig. 3, Table 3). Patients treated with both neoadjuvant chemotherapy and neoadjuvant radiation therapy had better OS than did patients who received neoadjuvant chemotherapy only (37.9 versus 25.9 months *p* = 0.024) (Fig. 4). There were no significant differences in 30-day mortality (0% in the neoadjuvant treatment group versus 1.2%; *p* = 0.388), 3-day unplanned readmission (3.5% in the neoadjuvant treatment group versus 5.4%; *p* = 0.43) or 90-day mortality (3.7% in the neoadjuvant treatment group versus 2.6%; *p* = 0.4) according to the neoadjuvant chemotherapy status.

**Table 3:** Overall survival

	Median OS with NAT (95%CI)	Median OS without NAT (95%CI)	*p*-Value
Tumor grade: poorly/undifferentiated	30.9 (19.5–42.2)	15.9 (11.8–20.1)	**0.006**
Clinical T4 tumors	23.9 (4.2–37.8)	12.1 (5.6–NA)	0.902
Metastatic disease	30.9 (3.1–58.6)	37.4 (18–56.8)	0.258
Tumor size > 20 mm	30.3 (21.4–39.1)	56 (39.8–72.2)	0.169

Bold text in the *p*-value column indicates statistical significance

*OS* Overall survival, *NAT* neoadjuvant therapy, *CI* confidence interval

## Discussion

Rectal NETs are relatively rare and data on the role of neoadjuvant chemotherapy for rNETs are scarce. The present study found that neoadjuvant chemotherapy had a survival benefit only for patients with poorly differentiated rNETs, which may have future implications in the management of this uncommon rectal neoplasm.

Given the rarity of rNETs, we opted to use the NCDB as it is a large-scale database with long-term follow-up, which is imperative when assessing the survival benefit of any therapy. Advanced NETs are known to have a worse prognosis [3] and the OS of these patients varies widely and depends on the mitotic rate and metastatic pattern [11, 12]. However, surgical resection still plays an important role in advanced rNETs [5, 6].

Age, sex, histologic grade (including differentiation, proliferation, and mitotic rate), tumor size, and stage at diagnosis are well-known prognostic factors for NETs [2, 3]. The results of the present study were consistent with this known prognostic factor as described in the literature. Specifically, we found that age, sex, tumor size, T stage, nodal status, tumor grade, and metastatic disease were all significantly associated with survival. On further analysis, only T4 stage, tumors > 2 cm, poorly differentiated tumors, and metastatic disease were independent prognostic factors. Unlike previous reports, [2, 13, 14] we did not find LN involvement to be an independent prognostic factor, although it was associated with worse survival on univariate analysis.

Currently, there is no standard chemotherapy protocol for NETs. However, the triple-drug combination regimen is generally used in patients with advanced NETs, although the response rate is dismal at only up to 30% [2].

There are very few studies on the outcomes of chemotherapy and radiotherapy for rNETs, particularly related to neoadjuvant treatment. Moreover, most of the current literature on this topic is of low quality and based on small-scale studies. A small investigation by Bireau et al. [15] showed that chemotherapy, with or without radiation therapy, had similar OS and progression-free survival as did surgery in nonmetastatic anorectal NETs. Modrek et al. [16] showed that radiotherapy improved OS for aggressive rNETs. In another investigation including only ten patients by Voong et al. [17] those with high-grade anorectal neuroendocrine carcinoma who completed chemoradiation and chemotherapy treatment over 13 years were assessed. Only three patients underwent surgery, and two of these patients received neoadjuvant treatment and achieved near pathological response. [17] According to the North American Neuroendocrine Tumor Society (NANETS) guidelines for well-differentiated NETs of the distal colon and rectum, chemotherapy is recommended for patients with clinically advanced aggressive tumors who lack other treatment options [6]. Chemotherapy and radiation therapy should be considered in patients with poorly differentiated extrapulmonary NETs [18] locoregional disease. In this setting, the benefit of surgery after completion of chemoradiation therapy is doubtful.

Based on multivariate analysis, four risk groups for poor survival of rNETs were identified, and the survival benefit of neoadjuvant chemotherapy was examined for each group. The survival benefit of neoadjuvant chemotherapy was evident only in poorly differentiated rNETs. This finding is concordant with a previous NCDB analysis by Erstad et al. [19] that found the administration of chemotherapy to be associated with a reduced risk of death in patients with high-grade rNETs, despite that only 6% of these patients achieved a complete pathological response.

Our study did not find any significant survival benefit in treating patients with large T4 tumors and metastatic disease with neoadjuvant chemotherapy. A previous case report aligns with our finding [20]. This report described the treatment of a female patient with high-grade rNET and liver metastasis with neoadjuvant chemotherapy, which was effective in downgrading the rectal tumor but did not result in significant changes in the size of the lesions at the rectum and liver [20]. Overall, the value of neoadjuvant therapy in metastatic disease is unclear with no unequivocal recommendations. Hormonal therapy, peptide receptor target radiotherapy (PRRT), liver direct therapy, radiotherapy, and systemic cytotoxic chemotherapy are all tools in the armamentarium of treating metastatic disease [5, 21].

The present study has several limitations, foremost of which is the retrospective nature of the NCDB. Nevertheless, since advanced rNET is a rare disease, the NCDB was needed to include a relatively high number of patients in the analysis. Furthermore, data related to the specificity of the chemotherapy and radiation regimen were not available, although we do know that these protocols were heterogeneous among patients. In addition, because the NCDB unfortunately does not include data on disease recurrence, we could not assess disease-free survival. The retrospective nature of the data limits our ability to account for all possible factors that influence the decision to give patients neoadjuvant chemotherapy.

## Conclusions

The role of neoadjuvant chemoradiotherapy treatment in rNETs has not been clearly established. We found that neoadjuvant treatment for patients with poorly differentiated tumors significantly improves their OS. We recommend that neoadjuvant treatment be considered for this specific patient group.

## Supplementary Information

Below is the link to the electronic supplementary material.

Supplementary file1 (DOCX 17 KB)

Supplementary file2 (DOCX 17 KB)

## Data Availability

Upon reasonable request to first author.
