# Supplementary material for: When is neoadjuvant chemotherapy indicated in rectal neuroendocrine tumors? An analysis of the National Cancer Database
Source: Tech Coloproctol. 2024 May 21;28(1):56. doi: 10.1007/s10151-024-02927-1 (PMC11108916; doi:10.1007/s10151-024-02927-1)
Supplement: Supplementary file 1 — Supplementary file1 (DOCX 17 KB) [file 10151_2024_2927_MOESM1_ESM.docx]

Supplementary table 1: Patients characteristics according to receipt of neoadjuvant chemotherapy

| **Factor** | **Group** | **No neoadjuvant chemotherapy** | **Neoadjuvant chemotherapy** | **p-value** |
| --- | --- | --- | --- | --- |
| n |  | 1493 | 146 |  |
| Mean age in years (SD) | | 56.29 (12.51) | 58.60 (12.44) | 0.033 |
| Sex (%) | Female | 751 (50.3) | 50 (34.2) | <0.001 |
|  | Male | 742 (49.7) | 96 (65.8) |  |
| Race (%) | American Indian | 9 ( 0.6) | 0 ( 0.0) | 0.044 |
|  | Asian | 130 ( 8.9) | 8 ( 5.5) |  |
|  | Black | 370 ( 25.3) | 24 ( 16.6) |  |
|  | White | 922 ( 63.0) | 112 ( 77.2) |  |
| Charlson-Deyo score (%) | 0 | 1189 (79.6) | 108 (74.0) | 0.028 |
|  | 1 | 226 (15.1) | 26 (17.8) |  |
|  | 2 | 58 (3.9) | 5 (3.4) |  |
|  | 3 | 20 (1.3) | 7 (4.8) |  |
| Clinical T stage (%) | 1 | 409 (69.0) | 11 (12.6) | <0.001 |
|  | 2 | 82 (13.8) | 13 (14.9) |  |
|  | 3 | 76 (12.8) | 53 (60.9) |  |
|  | 4 | 21 (3.5) | 10 (11.5) |  |
| Positive nodal status (%) | | 120 (14.4) | 55 (51.9) | <0.001 |
| Metastatic disease (%) | | 92 (9.1) | 17 (14.2) | 0.1 |
| Tumor size (%) | <10mm | 427 (46.5) | 7 (9.2) | <0.001 |
|  | 10-20mm | 199(21.7) | 9(11.8) |  |
|  | >20mm | 292 (31.8) | 60(78.9) |  |
| Tumor grade (%) | Low grade | 659(81.3) | 30 (31.2) | <0.001 |
|  | High grade | 152 (18.7) | 66 (68.8) |  |
| Neoadjuvant radiation (%) | | 10 ( 0.7) | 113 (79.0) | <0.001 |
| Positive surgical margins (%) | | 122 (8.4) | 28 (19.8) | <0.001 |

SD, standard deviation
