# Supplementary material for: When is neoadjuvant chemotherapy indicated in rectal neuroendocrine tumors? An analysis of the National Cancer Database
Source: Tech Coloproctol. 2024 May 21;28(1):56. doi: 10.1007/s10151-024-02927-1 (PMC11108916; doi:10.1007/s10151-024-02927-1)
Supplement: Supplementary file 2 — Supplementary file2 (DOCX 17 KB) [file 10151_2024_2927_MOESM2_ESM.docx]

Supplementary table 2: Patients characteristics according to tumor grade.

| **Factor** | **Group** | **Low Grade** | **High grade** | **p-value** |
| --- | --- | --- | --- | --- |
| n |  | 737 | 256 |  |
| Mean age in years (SD) | | 55.18 (11.87) | 62.87 (13.35) | <0.001 |
| Sex (%) | Female | 384 (52.1) | 112 (43.8) | 0.024 |
|  | Fale | 353 (47.9) | 144 (56.2) |  |
| Race (%) | American Indian | 4 ( 0.6) | 1 ( 0.4) | <0.001 |
|  | Asian | 76 (10.5) | 11 ( 4.3) |  |
|  | Black | 189 (26.2) | 40 (15.8) |  |
|  | White | 443 (61.4) | 197 (77.9) |  |
| Charlson-Deyo score (%) | 0 | 603 (81.8) | 186 (72.7) | 0.001 |
|  | 1 | 103 (14.0) | 43 (16.8) |  |
|  | 2 | 24 (3.3) | 19 (7.4) |  |
|  | 3 | 7 (0.9) | 8 (3.1) |  |
| Clinical T stage | 1 | 292 (74.1) | 16 (11.5) | <0.001 |
|  | 2 | 53 (13.5) | 25 (18.0) |  |
|  | 3 | 46 (11.7) | 76 (54.7) |  |
|  | 4 | 2 (0.5) | 21 (15.1) |  |
| Metastatic disease | | 47 (7.6) | 49 (21.7) | <0.001 |
| Positive nodal status | | 66 (12.5) | 93 (52.8) | <0.001 |
| Tumor size | <10 mm | 247 (49.3) | 11 (5.9) | <0.001 |
|  | 10-20mm | 113 (22.6) | 22 (11.9) |  |
|  | >20mm | 141 (28.1) | 152 (82.2) |  |
| Neoadjuvant chemotherapy (%) | | 30 (4.4) | 66 (30.3) | <0.001 |
| Neoadjuvant radiation (%) | | 28 (3.9) | 54 (22.0) | <0.001 |
| Positive surgical margins (%) | | 46 (6.3) | 64(25.5) | <0.001 |

SD, standard deviation
